# Modulation of the KEAP1-NRF2 pathway by Erianin: A novel approach to reduce psoriasiform inflammation and inflammatory signaling

**DOI:** 10.1515/biol-2025-1139

**Published:** 2025-07-11

**Authors:** Hongmei Yan, Gang Wang

**Affiliations:** Department of Dermatology, The Fourth People’s Hospital of Jinan, Jinan, 250031, China; Department of Dermatology, The Second Children & Women’s Healthcare of Jinan, Jinan, 271100, China

**Keywords:** psoriasis, inflammation, antioxidant, Erianin, KEAP1-NRF2 pathway

## Abstract

Psoriasis is a chronic, immune-mediated skin condition marked by excessive cell growth and inflammation. Current therapies frequently have limitations, such as side effects and insufficient efficacy, emphasizing the need for safer, more effective options. Erianin (ERN), a naturally occurring bioactive molecule produced from *Dendrobium chrysotoxum*, has anti-inflammatory and antioxidant characteristics, although its therapeutic potential in psoriasis has not been fully investigated. The purpose of this investigation was to look into the protective benefits of ERN against psoriasis-like skin inflammation utilizing laboratory-based cell models and an imiquimod-induced psoriasis animal model. Human keratinocytes were subjected to pro-inflammatory cytokines *in vitro* to simulate psoriasis disease, and cell survival and proliferation were measured. *In vivo*, mice given ERN for 6 days demonstrated a significant decrease in skin thickness, inflammatory cell infiltration, and overall histopathological alterations. ERN reduced pro-inflammatory substances (IL-6, IL-17, IL-23, IL-1β), TNF-α, COX-2, and inducible nitric oxide synthase, while increasing anti-inflammatory cytokine IL-10 and antioxidant-related molecules. Additionally, ERN stimulated the Kelch-like ECH-associated protein 1- nuclear factor erythroid 2-related factor 2 signaling pathway, which is essential for cellular antioxidant defense. The results presented here demonstrate that ERN reduces psoriasis-like inflammation by modifying immunological responses and increasing antioxidant protection, pointing to its potential as a viable therapeutic agent for psoriasis treatment.

## Graphical abstract

**Figure:**
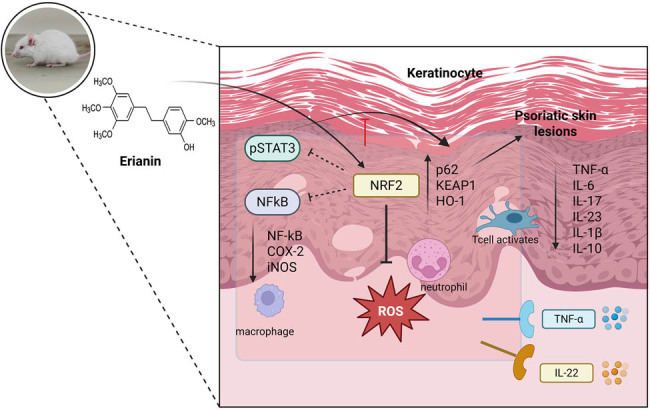

## 1 Introduction

Psoriasis affects millions of people around the world, a chronic, immune-driven skin disorder that has serious effects on both the body and the mind [1,2]. Psoriasis, which is distinguished by increased keratinocyte growth and chronic inflammation, mostly affects the epidermis, the outermost layer of the skin, and presents as red, scaly plaques [3,4]. This disorder includes intricate relationships between immune cells and keratinocytes at the molecular level, and the illness progresses due to the release of pro-inflammatory substances, including TNF-α, IL-17, and IL-23 [5,6]. Although the results of moderate-to-severe situations have benefited from biopharmaceutical drugs that target certain cytokines, these therapies are sometimes expensive and come with concerns of systemic immunosuppression [7]. Because of this, there is still a requirement for topical therapies that are safe, efficient, and affordable, particularly for individuals with mild-to-moderate illness [8,9,10].

The etiology of psoriasis appears to be linked to oxidative damage and inflammation signaling, according to the latest study, which has also identified potential treatment targets for these pathways [11,12,13]. Of them, the KEAP1-NRF2 pathway has attracted special attention because of its crucial role in shielding cells from oxidative damage and inflammation [14]. A gene nuclear factor erythroid 2-related factor 2 (NRF2), when triggered, coordinates the production of cytoprotective and antioxidant-related genes that aid in redox balance restoration and the suppression of inflammation-related signals [15,16]. Normally, NRF2 is appropriated in the cytoplasm and is destroyed by proteasomes under the control of its negative regulator, KEAP1 (Kelch-like ECH-associated protein 1) [17,18]. NRF2 parts from KEAP1 transfer into the nucleus and stimulate genes encoding anti-oxidants along with additional protective molecules in reaction to oxidative stress or certain chemical stimulants [19,20]. Thus, altering this system has become a viable treatment approach for inflammatory conditions, such as psoriasis, where immunological dysregulation and cellular oxidative stress are major causes [21,22].

Recent studies have underlined that psoriasis is more than just a skin disorder; it also has systemic effects on other essential organs. As an instance, psoriasis-like inflammation has been demonstrated to cause kidney dysfunction by upregulating NADPH oxidases and inducible nitric oxide synthase (iNOS), demonstrating that oxidative stress is a key contributor to organ damage beyond the skin [23]. Furthermore, psoriatic inflammation has been linked to hepatic inflammation as well as marked dysregulation in liver metabolism via IL-17A/IL-17 receptor signaling, emphasizing the importance of Th17 cytokines in extrapolating psoriatic pathologists to the livers [24]. Furthermore, modification of immunological signaling pathways, notably the IL-23/IL-17A axis, has emerged as a potential therapeutic target. Additionally, a BTK inhibitor was discovered to decrease imiquimod (IMQ)-induced psoriasis-like inflammation by modulating IL-23/IL-17A in innate immune cells [25], establishing a physiological link involving immune control and symptom alleviation. The results obtained highlight the systemic aspect of psoriasis and encourage more research into immune-modulatory treatments.

Natural products have a variety of pharmaceutical properties that may allow them to target key inflammatory factors, signaling pathways, and physiological activities associated with psoriasis [26,27]. The KEAP1-NRF2 pathway can be modulated by natural chemicals, providing a multi-targeted and frequently less hazardous therapy strategy [28,29]. Erianin (ERN), a naturally occurring isoflavone that comes from *Dendrobium chrysotoxum*, has shown anti-inflammatory, antioxidant, and anti-proliferative implications, among other pharmacological qualities [30,31,32,33,34,35]. According to preliminary research, by regulating proliferation and preserving the stability of the skin barrier, ERN can control keratinocyte behavior, something that is essential to the pathophysiology of psoriasis [34]. However, the precise ways in which ERN affects psoriatic inflammation specifically, via the KEAP1-NRF2 pathway, remain unclear. In the scenario of psoriasis, ERN is an intriguing option for more research because of the therapeutic potential of NRF2 induction in lowering the levels of oxidative and inflammatory pathways.

The persistence of the current investigation was to examine how ERN could affect psoriasiform inflammation by activating the KEAP1-NRF2 pathway. To evaluate ERN’s influence on cell proliferation and proinflammatory marker expression, we examined its effects on psoriatic skin cells *in vitro* using HaCaT cells treated with cytokines linked to psoriasis. To assess the *in vivo* effects of ERN on skin inflammation, antioxidant stress indicators, and important elements of the KEAP1-NRF2 pathway, we also used a mouse model of psoriasis produced by IMQ. Based on our research, ERN promotes a healthy intracellular context in psoriatic skin by lowering inflammation and promoting keratinocyte growth while also strengthening antioxidant defenses.

The therapeutic potential of ERN as a new topical medication for the treatment of psoriasis is highlighted by this study. ERN may provide a focused strategy for lowering oxidative stress, promoting skin health, and easing psoriasiform inflammation by modifying the KEAP1-NRF2 pathway. To deal with the demand for more effective and convenient choices for controlling mild-to-moderate psoriasis, this investigation offers important insights into the underlying mechanisms that govern the effectiveness of ERN and establishes the groundwork for its progress as a topical drug.

## 2 Materials and methods

### 2.1 Cell culture

The human differentiated keratinocytes (HaCaT; Procell, Wuhan, China) were cultivated in a moist incubator at 37°C and 5% CO_2_ using DMEM supplied with 10% FBS and 1% penicillin-streptomycin. At 37°C and 5% CO_2_, an *in vitro* cell model for psoriasis was created by inducing 10 ng/mL TNF-α in a logarithmic growth phase of HaCaT cells [36], for 48 h.

### 2.2 CCK8 assay

To ascertain the antiproliferative effect of ERN on HaCaT, the CCK-8 reagent was used. For a brief duration, HaCaT cells were seeded at a rate of 5 × 10^3^ cells/well in 96-well dishes, administered ERN at various doses (0, 5, 10, 20, 40, and 80 μM) for 24 h, and then maintained with CCK-8 reagent for 1 h at 37°C in the dark. ERN was administered to HaCaT cells at doses ranging from 5 to 20 μM following a 48 h TNF-α treatment at 37°C and 5% CO_2_. Utilizing a Microplate Reader, the absorption coefficient of the assessments was measured at an apparent wavelength of 450 nm.

### 2.3 EdU assessment

The HaCaT cells were cultivated in 24-well plates and exposed to varying doses of combined TNF-α and ERN. As directed by the manufacturer, the cell proliferation EdU (5-ethynyl-2′-deoxyuridine) assay kit (Thermo Fisher Scientific, Catalog No. C10337) was utilized to measure the proliferative growth rate of HaCaT cells. Immunofluorescence was used to evaluate EdU accumulation.

### 2.4 ELISA

ERN (5–10 μM) was applied to the TNF-α-persuaded HaCaT cells for 24 h. The ELISA kit (BlueGene Biotech, Catalog No. E03P0661) was then used to identify the various inflammatory cytokines (IL-6, IL-8, IL-22, and IL-1β) in the supernatant following the directions provided by the maker.

### 2.5 Reverse transcription-polymerase chain reaction (RT-PCR) analysis

After seeding HaCaT keratinocytes in 12-well plates, they were either triggered using TNF-α (10 ng/mL) or without it for 2 h after being treated with ERN (5, 10, and 20 μM) or not for 24 h. RNA was collected from cells and used for qRT-PCR investigation of Keratin 1 (KRT1) and Keratin 6 (KRT6) mRNA levels. As directed by the supplier, the TRIzol^TM^ reagent was used for extracting total RNA from mouse skin tissue or HaCaT keratinocytes. 2 µg of total RNA was used to create the first strand of cDNA using the cDNA Synthesis Kit (Servicebio, Catalog No. G3331). Using the SYBR Green PCR Master Mix kit (Thermo Fisher Scientific, Catalog No. 4309155), GAPDH was used as an internal control gene, and the relative mRNA expression levels of the target genes were calculated using the 2^−ΔΔCt^ method. This method allowed for the comparison of gene expression across different experimental groups. All reactions were performed in triplicate, and data were analyzed using the CFX96 Touch™ Real-Time PCR Detection System software. To target the genes in HaCaT keratinocytes, the following primer sequences were used (Table 1).

**Table 1:** Forward and reverse primer sequencing for RT-PCR analysis

Genes	Forward Primer (5′ → 3′)
	Reverse Primer (5′ → 3′)
KRT6	F 5′-GGGTTTCAGTGCCAACTCAG-3′
	R 5′-CCAGGCCATACAGACTGC GG-3′
KRT1	F 5′-CTTTTCTGCTGTTTCCCAATGAA-3′
	R 5′-GGAAAGAACAAAGCAGGG TCATAG-3′
GAPDH	F 5′-CACATGGCCTCCAAGGAGTAA-3′
	R 5′-TGAGGGTCTCTCTCTTCCTCT TGT-3′

### 2.6 Animal studies

All experiments on animals were conducted on male BALB/c mice in optimal condition, evaluating between 24 and 28 g at 5 weeks of age. The mice were housed in the animal house facility of the Department of Dermatology, The Second Children and Healthcare of Jinan, China, for approximately 7 days to allow them to become used to the laboratory environment (CPCSEA No: LL2024250001). A regular pellet meal and unlimited access to clean water were provided to the animals. For the duration of the investigative protocol, animals were kept in typical laboratory settings with a 12 h light/dark cycle, a temperature of 22 ± 2°C, and a moisture content of 40–70%. All that was possible has been taken to minimize the discomfort that the animals went through and to use only a few in totals for the assessment.


**Ethical approval:** The research related to animal use has been complied with all the relevant national regulations and institutional policies for the care and use of animals, and has been approved by the Animal Care and Use Committee of the Department of Dermatology, The Second Children and Healthcare of Jinan, China (Approval No: CPCSEA No: LL2024250001).

### 2.7 Fabrication of an experimental psoriasis system and therapy strategy in mice activated by IMQ

An animal model of IMQ-induced testing psoriasis was created [37], by topically treating mice with IMQ 5% (w/w) for 6 days in a row (days 1–6). A 2.5 cm × 2.5 cm section of every mouse’s dorsal skin was shaved to remove mouse hairs. IMQ and ERN were not applied topically to the control group. Based on day 1 to day 6, all the animals aside from the control group, were topically administered with commercially accessible IMQ, at an administration of 62.5 mg lotion on their smooth-shaven rear skin (50 mg of IMQ lotion), every morning. This equates to a daily dose of 3.125 mg of the effective IMQ. For the conventional clobetasol group of controls, 40 mg of commercially accessible clobetasol cream (0.05% w/w) was administered topically on all the animals’ trimmed back skin (equivalent to 32 mg of clobetasol cream) every day, resulting in a daily supply of 20 μg of the influential substance.

External administration of ERN was performed in an anionic suspension base containing 100 mg at three distinct concentrations of 0.5, 1, and 2% w/w per animal. Each animal's shaved dorsal skin was topically treated with 100 mg of the produced anionic emulsifying cream containing ERN (0.5, 1, and 2% w/w) for six days in a row. This amounts to 80 mg of ERN cream each application. This equates to a daily allowance of 0.5, 1, and 2 mg of the ERN dose for each animal, Accordingly, every aspect concentration was directed at 12 h intervals after the use of IMQ for 6 successive days, except for control and IMQ control. Six groups of six mice were chosen at random from among the mice (*n* = 6).

### 2.8 Experimental groups

Group I: control (without IMQ and ERN)

Group II: IMQ control (IMQ alone; without ERN)

Group III: IMQ + 0.05% w/w Clobetasol (standard drug)

Group IV: IMQ + 0.5% w/w ERN (IMQ + ERN 0.5% w/w)

Group V: IMQ + 1% w/w ERN (IMQ + ERN 1% w/w)

Group VI: IMQ + 2% w/w ERN (IMQ + ERN 2% w/w)

The psoriasis area and severity index (PASI), which is rated using successive days’ aggregate scores for scaling, skin redness, and thickness, was used to track the infection’s progression. Day 7 saw the aseptic collection of the animals’ blood, spleen, skin, and ears, as well as their CO_2_ asphyxia death. The sections of the epidermis and ear tissues that were extracted were preserved in 10% formalin, and for later analysis, the remaining tissues were stored at −80°C. Blood was extracted to determine the hematological and biochemical features. The skin tissue in the IMQ-treated region provided the expected parameters for the entire investigation.?A3B2 tpb -13pt?>

### 2.9 Evaluation of the PASI

Animals were examined from day 1 to day 6 to calculate the PASI (for instance, following IMQ administration). The PASI was rated by the days that followed. On a scale of 0–4, erythema, skin thickness, and scales were rated distinctly as follows: Erythema: 0 (None), 1 (Weak), 2 (Moderate), 3 (Severe), 4 (Very severe), Skin thickness: 0 (None), 1 (Slightly thickened), 2 (Moderately thickened), 3 (Markedly thickened), 4 (Very markedly thickened), and Scales: 0 (None), 1 (Few scales), 2 (Moderate scales), 3 (Numerous scales), and 4 (Very numerous scales). The overall PASI score, which ranged from 0 (no inflammation) to 3 (severe inflammation), was computed by adding the distinct scores for erythema, skin thickness, and scales for every animal. This score was used to measure the level of inflammation involved with the psoriasis-like erythema on each day of the examination [38]. An aerospace digimatic micrometer Screw gauge was used to measure the thickness of the ear folds. On different days, the dorsal skin photos were captured to investigate the complete macroscopic structure in IMQ-induced animals.

### 2.10 Spleen to the mass ratio of the body

The animals’ weights were recorded before their killing and continued throughout the conclusion of the research, till the moment when the spleens were removed, cleansed, and evaluated. The spleen masses were regulated using body weight to compute the organ index. The results that were obtained were expressed in grams per gram.

### 2.11 Determining parameters for hematology

On the seventh day, blood from every testing animal was collected via the retroorbital plexus and placed into containers containing the anticoagulant substance EDTA. To ascertain the hematological features, a blood cell counter was employed. Hematological cell counts, measured in WBC, have been defined as (×10^3^ cell/μL).

### 2.12 Histopathological evaluation

Before being embedded in paraffin, the rear skin of the assassinated animals had been eliminated and treated in 4% paraformaldehyde. H & E staining was applied to the paraffin-embedded portions after they had been cut to a thickness of 5 μm for histological assessment.

### 2.13 Measuring oxidative stress markers and antioxidant defenses

Commercial kits for malondialdehyde (MDA) (Catalog No. A003-1), NO (Catalog No. A012-1-1), glutathione (GSH) (Catalog No. A006-2), glutathione s-transferases (GST) (Catalog No. A004), total superoxide dismutase (SOD; A001-1), and catalase (CAT; A007-1), Vitamin C (Catalog No. A009), and thiobarbituric acid reactive substances (TBARS) (Catalog No. A003-1) were purchased from Nanjing Jiancheng Bio Ins (Nanjing, China). Protein samples from skin tissues or cell supernatants were isolated and utilized to evaluate the contents of the aforementioned oxidative stress components using commercially available kits following the manufacturer’s instructions.

### 2.14 Myeloperoxidase (MPO) activity assay

MPO activity was measured using Bradley et al.’s [39] methodology. MPO activity is an indication of neutrophils’ infiltration of inflammatory cells in the dermis layer of psoriasis. Skin tissues were dissolved in ice-cold 0.1 M potassium phosphate buffer (pH 6.5) with 0.5% hexadecyltrimethylammonium bromide and 10 mM EDTA. Tissue homogenates have been centrifuged at 13,100 × *g* for 20 min at 4°C. An aliquot of the supernatant (0.1 mL) was mixed with 2.9 mL of 50 mM phosphate buffer containing 0.167 mg o-dianisidine hydrochloride and 0.0005% hydrogen peroxide. The absorbance change over 5 min was measured at 460 nm. The data were presented as U/g of tissue.

### 2.15 Western blot analysis

BCA kit (Sigma-Aldrich, Catalog No. 71285-M) was used to homogenize and measure the cellular and tissue homogenates that are used to measure the protein fractions. Polyvinylidene fluoride membrane were equally loaded with the protein samples after they had been separated on 10% SDS polyacrylamide gels. The membranes were treated with primary antibodies overnight at 4°C following a 1 h incubation period with 5% bovine serum albumin. All primary (Rabbit IgG) and secondary antibodies were purchased from Cell Signaling Technology, USA, and were employed at the specified dilutions. The expressions of Nf-κB p65 (1:500), NRF2 (1:1,000), COX (1:1,000), iNOS (1:1,000) p62 (1:1,000), and KEAP1 (1:1,000) were studied. β-actin (1:1,000) was used as a housekeeping protein for whole-tissue extracts, and Lamin B (1:1,000) was used as a housekeeping protein for nuclear extracts. Horseradish-peroxidase (HRP)-conjugated anti-mouse or anti-rabbit antibodies (1:5,000) were used as secondary antibodies. Digital imaging devices used ECL to identify protein expression on blots after they were treated for 1 h with secondary antibodies coupled with HRP. The western blot findings’ intensity was examined using ImageJ software.

### 2.16 Examining data statistically

All statistical analyses were performed using GraphPad Prism software. Data were expressed as mean value ± standard error of the mean (SEM). To assess statistical significance, one-way analysis of variance was used, followed by Tukey’s *post hoc* test to compare differences between groups. A *p*-value of less than 0.05 was considered statistically significant.

## 3 Results

### 3.1 Effect of ERN on keratinocyte viability and TNF-α-induced proliferation

**Figure 1:**
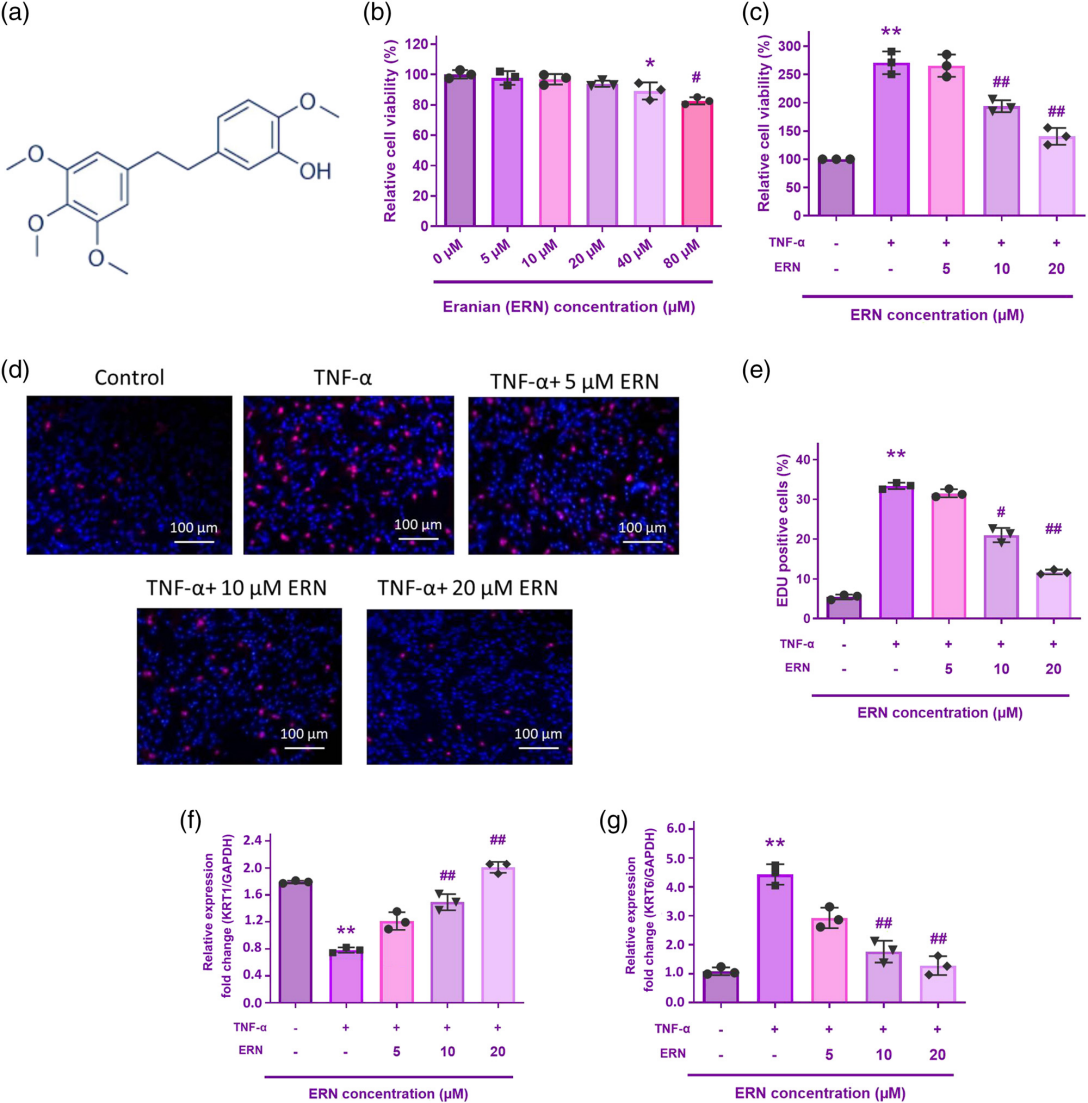
The impact of ERN on TNF-α-stimulated HaCaT cell proliferation. (a) ERN molecular structure. (b) and (c) Cell viability was assessed using the CCK-8 assay for 24 h. (d) The EdU assay was utilized to quantify the proliferation rate. (e) Quantification of EdU-positive cells in HaCaT cells treated with TNF-α (10 ng/mL) and increasing concentrations of ERN (5, 10, and 20 μM) for 24 h. The bar graph represents the percentage of proliferating (EdU-positive) cells. Data are expressed as mean value ± SD (*n* = 3). (f) and (g) KRT6 and KRT1 mRNA levels were analyzed by qRT-PCR. The internal reference is GAPDH. **P < 0.01 vs control. ^##^ *P* < 0.01 vs TNF-α group. Data are expressed as mean value ± SD.

The molecular structure of ERN is shown in Figure 1a. The effects of ERN on human keratinocyte viability were assessed using the CCK8 assay. Following ERN therapy, no discernible toxicity was seen at dosages lower than 40 μM (Figure 1b). The inhibitory effect was particularly pronounced at higher concentrations (above 40 µM), suggesting that ERN exerts cytotoxic effects on keratinocytes at elevated doses. Thus, for the following investigations, keratinocytes were exposed to 5, 10, and 20 μM ERN.

To confirm the impact of ERN on TNF-α-induced proliferation of HaCaT cells, the growth of HaCaT cells was measured using the CCK-8 and EdU assays. Proliferating cells were triggered by TNF-α, but this effect was mitigated by ERN administration at 5, 10, and 20 μM (Figure 1c). The findings of EdU staining verified that the proliferation of HaCaT cells was significantly increased by TNF-α therapy. Intriguingly, ERN reduced the rate of proliferation brought on by TNF-α induction in a manner that was dose-related (Figure 1d).

KRT6 is recognized as a marker of hyperproliferation in psoriatic keratinocytes. qRT-PCR analysis demonstrated that TNF-α exposure significantly upregulated KRT6 mRNA expression (Figure 1e), indicating increased keratinocyte proliferation. Conversely, KRT1, a marker of keratinocyte differentiation, was significantly downregulated following TNF-α treatment (Figure 1f). Cotreatment with ERN effectively reduced KRT6 expression in a dose reliant on the way, suggesting that ERN inhibits TNF-α-persuaded keratinocyte hyperproliferation. However, KRT1 expression was also increased upon ERN treatment, which implies that ERN restores keratinocyte differentiation. These findings suggest that ERN exerts an anti-proliferative effect on TNF-α-stimulated keratinocytes, supporting the successful establishment of an *in vitro* psoriasis model.

### 3.2 ERN reduces inflammatory cytokine stimulation

**Figure 2:**
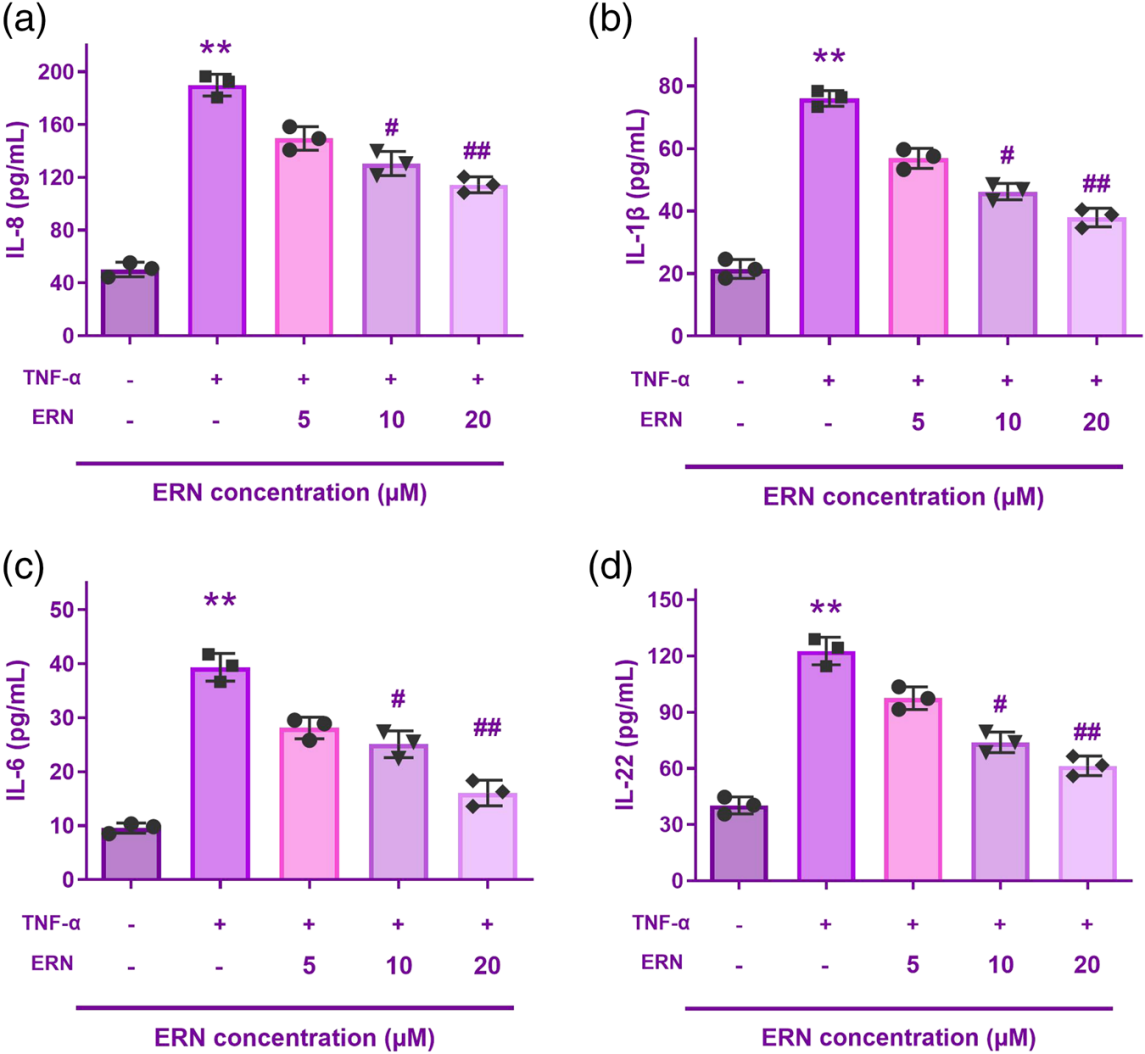
ERN reduced the inflammation of HaCaT cells caused by TNF-α. (a–d) Interleukin-8 (IL-8), IL-1β, IL-6, and IL-22 levels are all reduced by ERN. **P < 0.01 vs control. ^##^ *P* < 0.01 vs TNF-α group. Data are expressed as mean value ± SD.

Measurement of various inflammatory cytokines linked to the emergence of inflammation, including IL-6, IL-8, IL-22, and IL-1β, was done using ELISA to ascertain the effect of ERN (5, 10, and 20 μM) on TNF-α-induced inflammation of HaCaT cells (Figure 2). According to the ELISA results, the TNF-α monotherapy group’s expressions of inflammatory factors were considerably higher than those of the untreated group. However, in a dose-dependent manner, ERN significantly decreased them. In a dose-responsive way, the data showed that ERN reduced the inflammation of HaCaT cells caused by TNF-α.

To confirm its promise as a treatment, we expanded our research to include an *in vivo* psoriasis model. We used an IMQ-induced psoriasis approach, which particularly resembles human psoriasiform inflammatory processes, to assess ERN’s effectiveness in a physiologically appropriate environment. The implications of ERN on oxidative stress, inflammatory release of cytokines, and keratin hyperplasia can be evaluated *in vivo* using this paradigm.

### 3.3 Therapeutic impact of ERN on psoriasis severity, body weight, and spleen index

**Figure 3:**
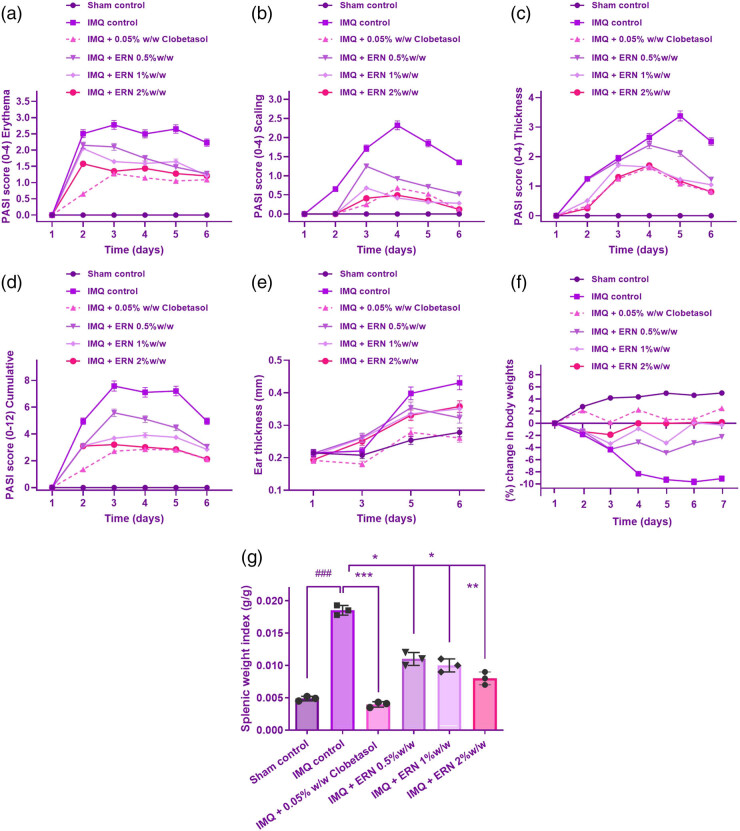
Effect of ERN on IMQ-induced psoriasiform severity in mice. ERN treatment significantly alleviated psoriatic skin changes in mice after 6 days, resembling human psoriasis. The severity of psoriasiform lesions was evaluated using the PASI based on erythema (redness), scaling, and skin thickness, scored from 0 to 4. The following parameters were assessed: (a) Erythema, (b) scaling, (c) thickness, (d) cumulative PASI score, (e) ear thickness, measured using a screw gauge on days 1, 3, 5, and 6, (f) changes in body weight induced by IMQ and its modulation by ERN treatment, (g) spleen mass, calculated as the spleen-to-body weight ratio, comparing ERN-treated and IMQ control groups. Results are expressed as mean value ± SEM (*n* = 8). ^###^ *p <* 0.001; ^##^ *p <* 0.01, ^#^ *p <* 0.05 compared with the sham control group; *p < 0.05, **p < 0.01, and ***p < 0.001 compared with the IMQ alone treated group; ns, non-significant.

To determine the degree of psoriasis lesions in the IMQ-induced psoriasis model, the PASI was measured regularly from day 1 to day 6 following IMQ administration. Erythema, skin thickness, and scaling were individually recorded on a scale from 0 to 4: 0, none; 1, weak; 2, modest; 3, noticeable; and 4, very noticeable. The scores for each parameter were then summed to obtain a composite PASI score, which could range from 0 (no inflammation) to 12 (severe inflammation). Here are the specific findings based on the PASI and other measures such as erythema, skin thickness, scaling, composite PASI score, and macroscopic morphology (Figure 3).

IMQ administration induced a progressive increase in erythema from day 1, peaking around day 4 and remaining at a high level through day 6. Animals treated with ERN showed an important drop in erythema related to the IMQ-only group, with erythema scores in the ERN-treated group remaining 1–2 points lower throughout the treatment period (Figure 3a).

IMQ-induced scaling became evident by day 2 and intensified through day 6 (Figure 3b). In the IMQ group, scales were thick and widespread, with scores reaching 3–4 by day 4. ERN-treated animals showed a marked reduction in scaling, with scores approximately 1–1.5 points lower than the IMQ-only group. On Day 6, the 1% ERN group had marginally higher PASI scaling scores than the 0.5% group. These data point to a potential plateau or nonlinear connection in ERN’s therapeutic impact at increasing dosages. By the end of the observation period, scaling was significantly reduced in ERN-treated animals, suggesting ERN’s effectiveness in controlling hyperkeratosis.

Measurement of skin thickness using an Aerospace digimatic micrometer screw gauge demonstrated that the IMQ-treated animals developed substantial epidermal thickening by day 2, which continued to increase until day 6. ERN treatment significantly attenuated this increase, with skin thickness measurements consistently showing a lower degree of thickening compared to the IMQ-only group. By day 6, ERN-treated animals displayed skin thickness scores that were approximately 30–40% lower than untreated IMQ animals (Figure 3c).

The overall PASI score, obtained by summing the erythema, skin thickness, and scaling scores, provided a cumulative measure of psoriasis severity. In IMQ-only animals, the PASI score increased from an average of 2 on day 1 to around 10 by day 6, indicating severe psoriasiform inflammation. ERN treatment consistently reduced the PASI score across all days, with a final average PASI score of approximately 5 by day 6. This reduction in PASI score indicates a substantial alleviation of psoriasis-like symptoms with ERN treatment (Figure 3d). ERN considerably decreased and maintained the ear’s IMQ-induced epidermal thickness, as seen in Figure 3e. Also, it was observed that the animals in the common drug group demonstrated a reduction in their psoriatic symptoms.

Macroscopic morphology of the dorsal skin on different days revealed visible differences between IMQ-only and ERN-treated animals (Figure S1). IMQ-treated animals exhibited progressively more pronounced erythema, thickening, and scaling over time. In contrast, the ERN-treated animals maintained relatively smooth and less inflamed skin, with a reduced severity of scales and erythema, supporting the quantitative PASI findings.

Body weight measurements were made daily to analyze the consequences of ERN therapy (0.5, 1, and 2% w/w) and IMQ treatment. The IMQ-treated category’s body weight steadily dropped for 6 days, which was probably due to systemic inflammation and stress caused by psoriasis-like skin lesions. At all dosages (0.5, 1, and 2%), ERN treatment reduced IMQ-induced weight loss, with higher doses showing a more pronounced prophylactic effect (Figure 3f). There existed no notable distinctions between the ERN-treated category and the untreated group, indicating that ERN therapy had no adverse systemic effects.

The weight of every animal’s spleen was divided by its total weight to determine the spleen-to-bodyweight ratio, often known as the organ’s index. In the IMQ-only group, the organ index was notably elevated, with a mean increase of approximately 40–50% compared to control animals. This elevated organ index reflects the systemic inflammatory response triggered by IMQ treatment.

In contrast, ERN treatment led to a substantial decline in the organ index associated with the IMQ-only group. ERN-treated animals displayed an organ index that was approximately 20–30% lower than the IMQ group, indicating mitigation of spleen enlargement and suggesting an anti-inflammatory effect of ERN on systemic immune responses (Figure 3g). Statistical comparisons between groups revealed that the organ index in the IMQ-only group was significantly higher than in the control group (*p* < 0.05). ERN treatment significantly condensed the organ index associated with the IMQ-only group (*p* < 0.05), indicating that ERN treatment attenuated the systemic inflammatory response. The enhanced immunological activity brought on by psoriasiform inflammation is consistent with the higher organ index in the IMQ-only group. The spleen-to-bodyweight ratio decreased in the group that received ERN treatment, indicating that ERN has an immune-regulating impact that lowers the level of systemic inflammation. These results indicate that ERN may help to control not only local skin inflammation but also the broader immune activation associated with psoriasis.

### 3.4 Effect of ERN on oxidative stress markers in IMQ-induced skin inflammation in animal tissue

**Figure 4:**
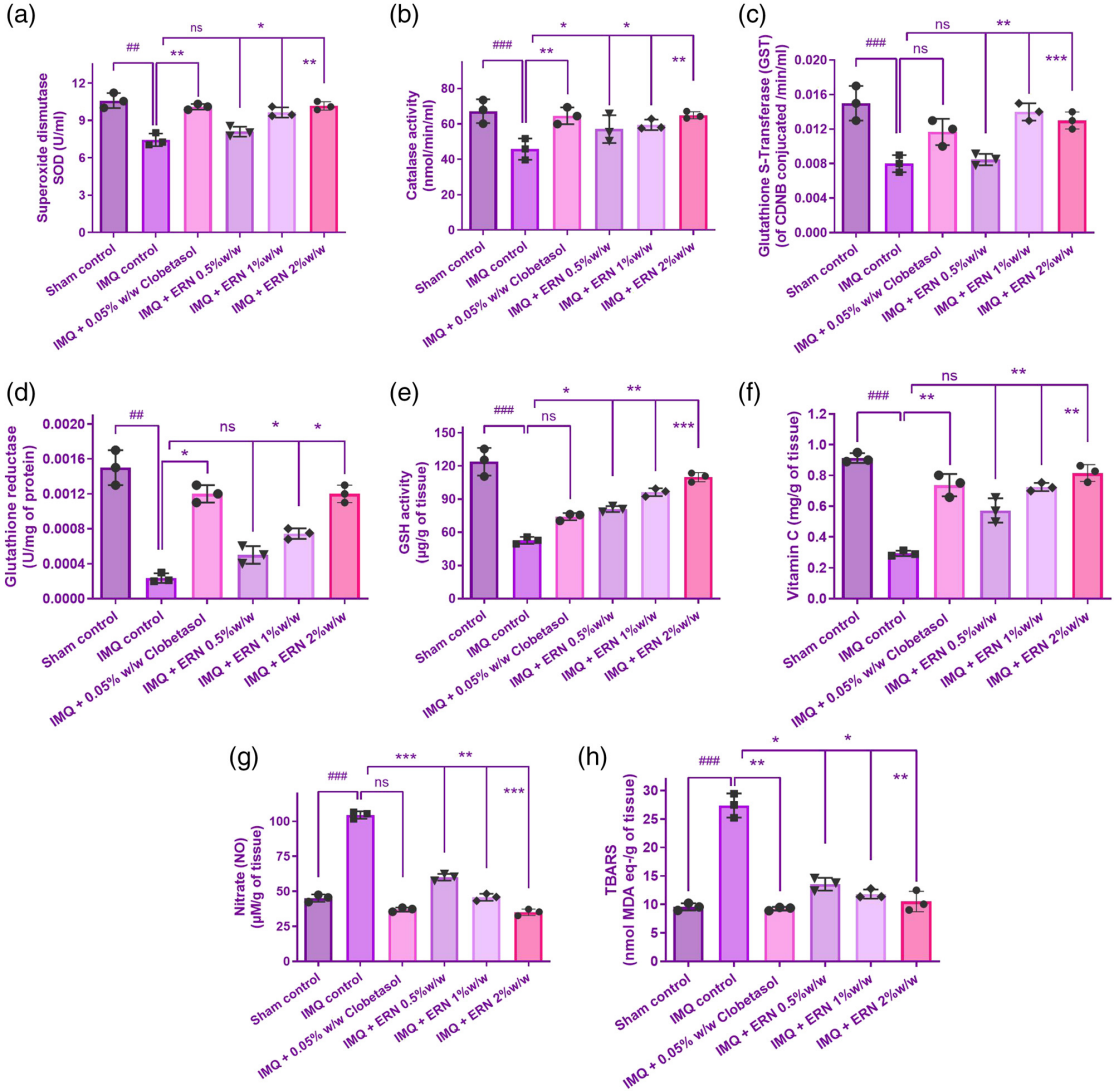
Effects of ERN treatment on antioxidant and oxidative stress markers in IMQ-induced psoriasis- skin tissue. (a)–(h) Biochemical analysis of antioxidant enzyme activity and oxidative stress markers in all experimental groups. Results are presented as mean values ± SEM (*n* = 6). ^###^ *p <* 0.001, ^##^ *p <* 0.01, ^#^ *p <* 0.05 compared with the sham control group; *p < 0.05, **p < 0.01, and ***p < 0.001 compared with the IMQ alone treated group; ns, non-significant.

Figure 4 shows that treatment with IMQ alone substantially exacerbated oxidative stress, as demonstrated by elevated levels of MDA and nitric oxide (NO), as well as a substantial decline in antioxidant defense markers such as GSH, SOD, CAT, and vitamin C when compared to the control group. These data demonstrate that IMQ weakens the antioxidant barrier, resulting in increased oxidative damage.

On the other hand, ERN therapy dramatically reduced the oxidative damage caused by IMQ. This was demonstrated by reduced MDA and NO levels, as well as the restoration of antioxidant indices such as GSH, SOD, CAT, and vitamin C activities (Figure 4a–h). These findings imply that ERN improves the endogenous antioxidant defence system and might defend against IMQ-induced oxidative damage. The antioxidant enzyme levels (SOD and CAT) in the 2% ERN group were not substantially higher than those in the 1% group (Figure 4c and d). The results indicate an expected threshold or nonlinear connection in ERN’s therapeutic impact as doses increase. More research is needed to better understand the molecular processes behind ERN’s antioxidant capability.

### 3.5 Histopathological and clinical assessment of skin inflammation

**Figure 5:**
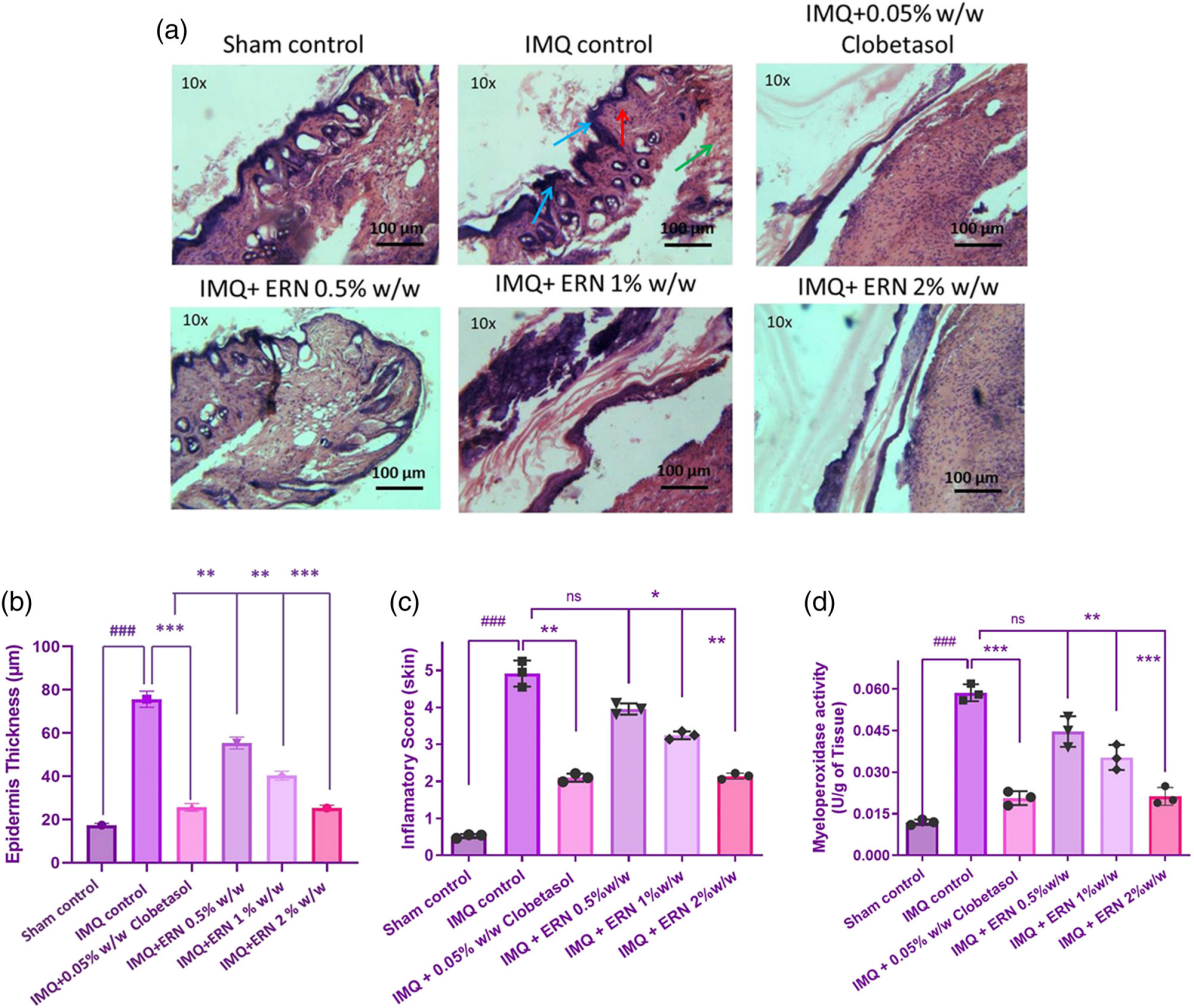
Impact of ERN treatment on histopathological alterations in IMQ tempted psoriasis. Representative H&E-stained photomicrographs of (a) skin tissue for all experimental groups. Histopathological assessments include blue arrows indicating hyperkeratosis, red arrows denoting acanthosis, and green arrows highlighting the infiltration of neutrophils. (b) Quantification of epidermal thickness from H&E-stained dorsal skin sections. Epidermal thickness was measured at five randomly selected sites per section using ImageJ software. IMQ-induced epidermal thickening was significantly reduced following ERN treatment in a dose-dependent manner. (c) Inflammatory score of the skin and (d) MPO activity in skin tissue. Blue arrows indicate hyperkeratosis, red arrows indicate acanthosis, and green arrows indicate infiltration of neutrophils in histopathology images. Data are expressed as mean values ± SD. *n* = 6 animals per group, experiments were repeated three times. ^###^ *p <* 0.001; ^##^ *p <* 0.01, ^#^ *p <* 0.05 compared with the sham control group; *p < 0.05, **p < 0.01, and ***p < 0.001 compared with the IMQ alone treated group; ns, non-significant.

Skin tissues were histologically examined with hematoxylin and eosin (H&E) staining to determine the therapeutic potential of ERN in IMQ-induced psoriatic mice (Figure 5a and b). Histological depiction of the IMQ-only group exhibited classic psoriatic characteristics such as extensive acanthosis, hyperkeratosis, parakeratosis, rete ridge elongation, and extensive dermal inflammatory infiltrates. In comparison, the control group’s skin had normal epidermal thickness and was free of inflammation.

ERN-treated groups (0.5, 1, and 2% w/w) showed dose-dependent enhancement in histopathological characteristics. The 2% ERN group had the greatest loss in epidermal thickness and immune cell infiltration, closely mirroring the skin architecture seen in the clobetasol-treated standard group. ERN treatment significantly reduced inflammatory severity, epidermal hyperplasia, and keratinocyte proliferation compared to IMQ alone (*p* < 0.001; Figure 5b). These refined histological data highlight the capacity of ERN to dramatically restore close to typical skin architecture in psoriatic mice. The combined visual and quantitative evaluations show strong evidence for ERN’s anti-psoriatic effectiveness.

### 3.6 Effect of ERN on skin tissue MPO

The skin tissue data showed that the IMQ monotherapy recipients had significantly greater MPO activity, a marker of neutrophil infiltration, than those who were not treated. As opposed to the IMQ monotherapy group of cures, MPO activities significantly decreased MPO levels during exposure to increased ERN doses (Figure 5c).

### 3.7 Determining parameters for hematology

**Figure 6:**
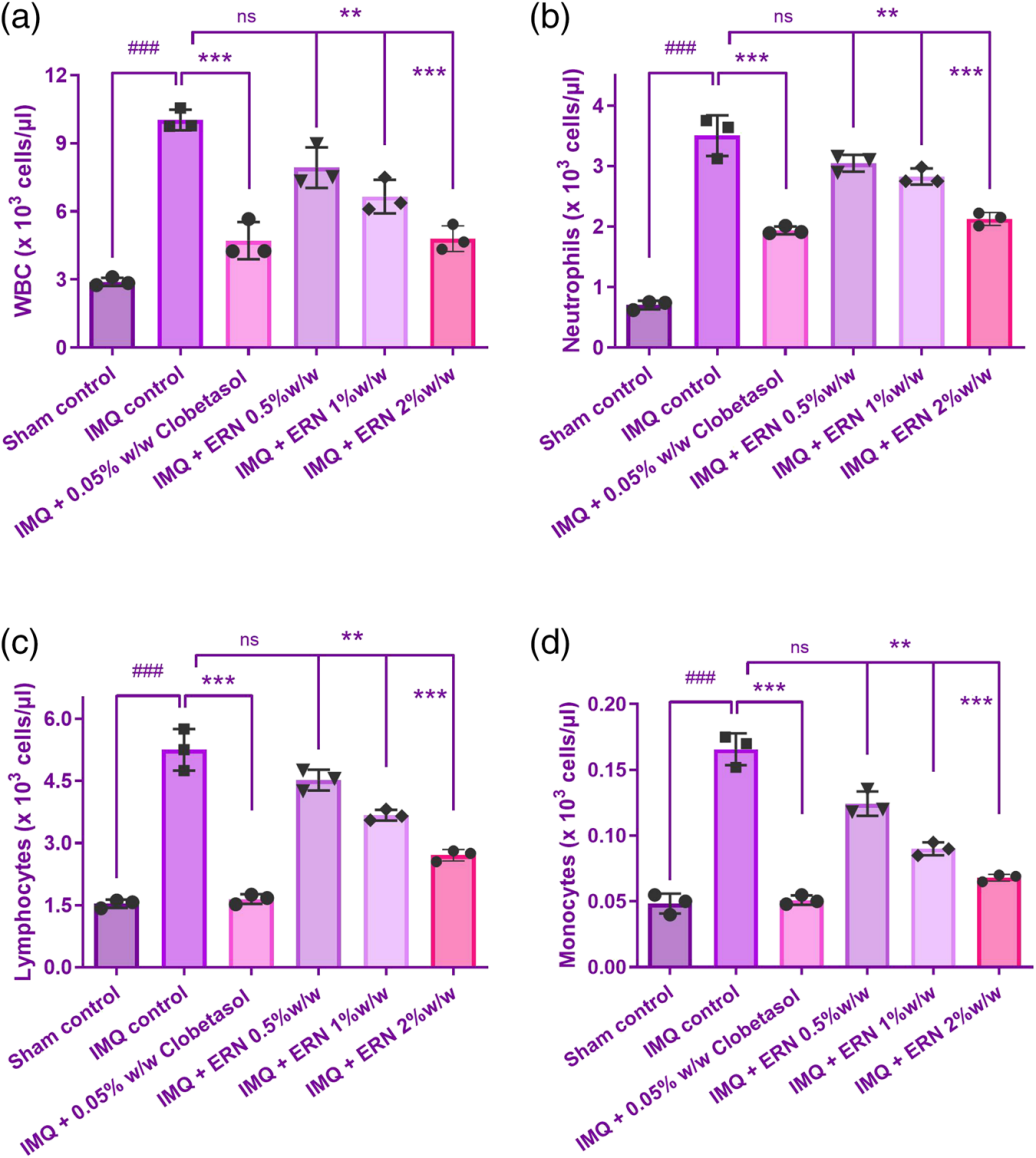
Effect of ERN treatment on hematological parameters in IMQ-exposure mice. Hematological analysis of whole blood from all experimental groups on day 7 post-treatment. (a) WBC count, (b) neutrophil count, (c) lymphocyte count, and (d) monocyte count. Results are expressed as mean value ± SEM (*n* = 6). ^###^ *p <* 0.001, ^##^ *p <* 0.01, and ^#^ *p <* 0.05 compared with the sham control group; *p < 0.05, **p < 0.01, and ***p < 0.001 compared with the IMQ alone treated group; ns, non-significant.

The blood cell count data revealed differential responses between the experiments, IMQ and control groups (Figure 6). Outcomes exhibited that the levels of WBC in the blood were noticeably greater in the IMQ individually treated group than those of the control group, indicating an inflammatory or immunological response to the therapy. Compared to the group that was obtaining IMQ alone, the treatment groups (IMQ + ERN) showed a substantial drop in WBC count (Figure 6a), which may indicate immunological suppression. The RBC count, hemoglobin, and hematocrit values did not significantly differ across groups, indicating that the experimental treatments did not affect these parameters. Consequently, our results demonstrate that ERN therapy effectively declined the quantity of WBCs in the distribution of whole blood, this has thus decreased the blood’s production of inflammatory chemicals, resulting in an anti-inflammatory impact.

The IMQ-only group showed a significant increase in neutrophils, monocytes, and lymphocyte count indicating an inflammatory response (Figure 6b–d). ERN treatment (0.5, 1, and 2% w/w) significantly modulated these changes in a dose-dependent manner. Neutrophil and monocyte levels were notably decreased, while lymphocyte counts were restored toward normal levels, suggesting an immunomodulatory and anti-inflammatory effect of ERN.

### 3.8 ERN-mediated modulation of pro-inflammatory and anti-inflammatory cytokines

**Figure 7:**
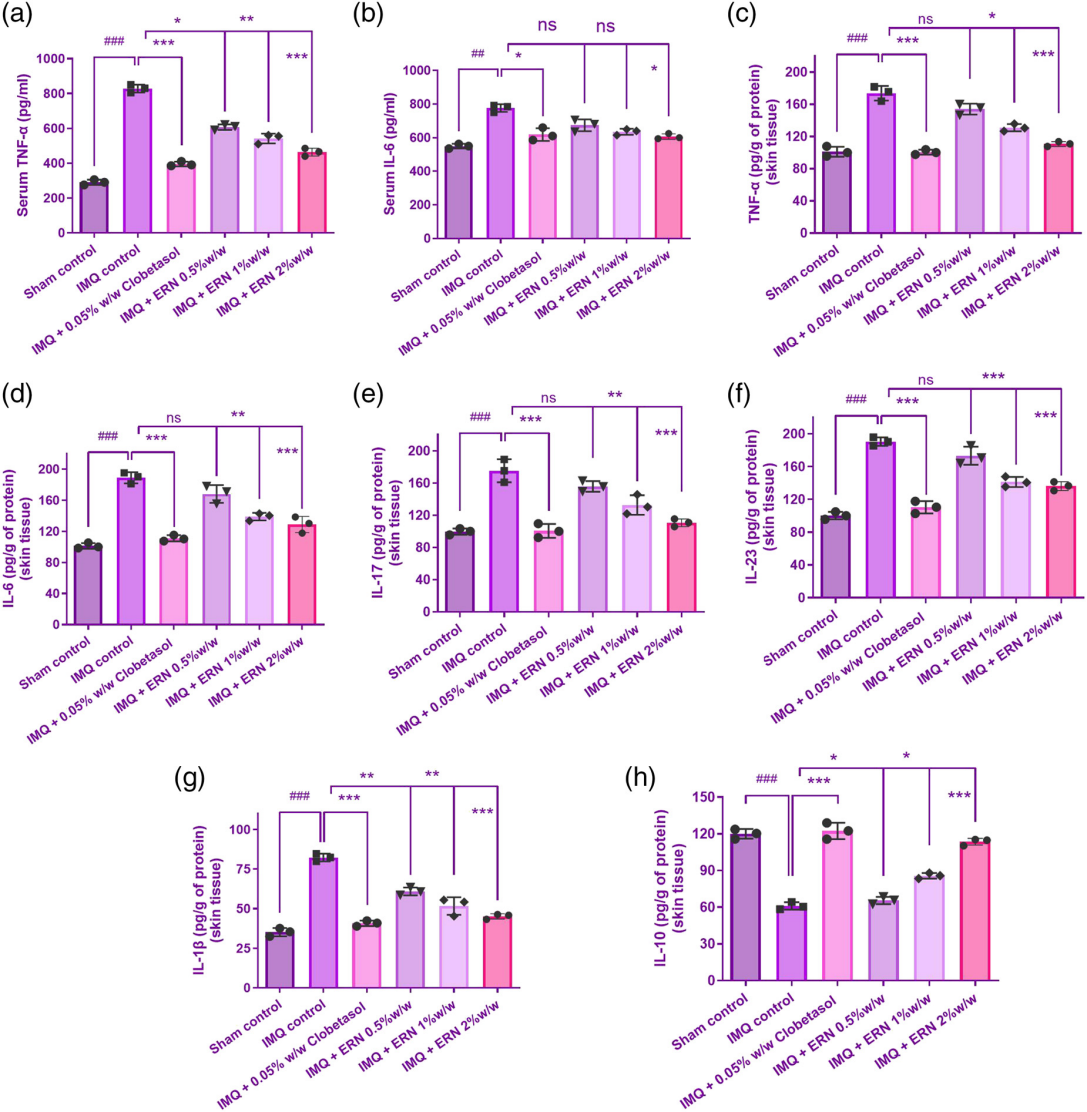
Proinflammatory and anti-inflammatory cytokine levels in IMQ-stimulated psoriatic skin tissue and serum. (a) TNF-α, (b) IL-6 levels, (c) TNF-α, (d) IL-6, (e) IL-17, (f) IL-23, and (g) IL-1β levels in mice skin tissue at 7 days post-ERN treatment. (h) IL-10 level in mouse skin tissue with ERN treatment, representing anti-inflammatory response. Cytokine levels were quantified using ELISA kits. The mean value ± SEM is used to present the data (*n* = 6). ^###^ *p <* 0.001, ^##^ *p <* 0.01, and ^#^ *p <* 0.05 compared with the sham control group; *p < 0.05, **p < 0.01, and ***p < 0.001 compared with the IMQ alone treated group; ns, non-significant.

The proportions of pro-inflammatory (TNF-α, IL-6, IL-17, IL-23, and IL-1β) and anti-inflammatory (IL-10) cytokines were assessed in the serum and cutaneous tissue of psoriatic animals produced by IMQ to assess the immunomodulatory impact of ERN in psoriasis-like inflammation. Pro-inflammatory cytokines were significantly reduced in the skin tissue and serum in dissimilarity to the subjects treated with IMQ (Figure 7a–g), suggesting that ERN effectively modulates systemic and localized inflammation in psoriasis.

The levels of anti-inflammatory cytokines elevated after ERN treatment, especially IL-10, and these had elevated levels in the treated animals’ blood and epidermis (Figure 7h). This supports ERN’s promise as a treatment for psoriasis by indicating that it encourages the skin to respond in a way that is less inflammatory.

### 3.9 Impact of ERN on NF-κB signaling

**Figure 8:**
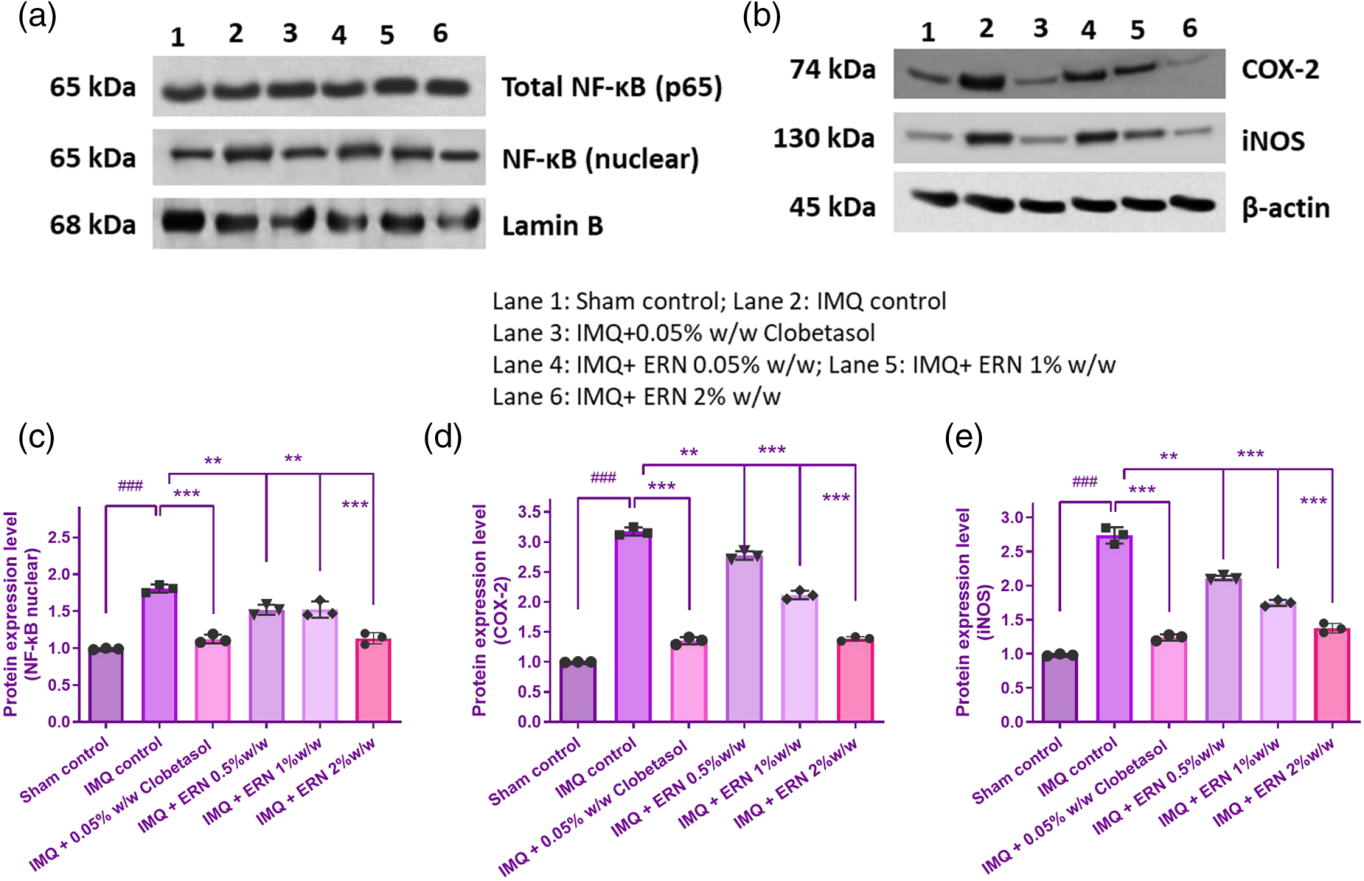
Impact of ERN therapy on the NF-κB signaling pathway in skin tissue produced by IMQ. The appearance levels of p65, COX-2, and iNOS in the skin tissue of IMQ compared to ERN are shown in (a) and (b), an illustrative immunological blot analysis. β-actin and Lamin B served as the internal reference. (c)–(e) shows the band intensities measured concerning protein expression using Image J software analyses. The findings were expressed using the mean value ± SEM (*n* = 3). ^###^ *p <* 0.001 compared with the sham control group; **p < 0.01 and ***p < 0.001 compared with the IMQ alone treated group.

The present study assessed the expression profiles of key proteins involved in the NF-κB signaling pathway to evaluate the effect of ERN in IMQ-induced psoriatic skin. IMQ treatment markedly increased the total expression and nuclear localization of NF-κB p65, as demonstrated by Western blot (Figure 8a), indicating enhanced activation of the NF-κB pathway. In parallel, expression of downstream pro-inflammatory mediators iNOS and COX-2 was also significantly upregulated in IMQ-treated mice (Figure 8b). Treatment with ERN at concentrations of 0.5, 1, and 2% resulted in a dose-dependent reduction in nuclear NF-κB p65 expression (Figure 8c). Furthermore, this finding supported ERN’s function in preventing NF-κB-mediated inflammation by confirming lower nuclear translocation of NF-κB p65 in ERN-treated groups. This effect was accompanied by a substantial decrease in the expression of iNOS and COX-2 (Figure 8d and e), suggesting attenuation of the NF-κB-mediated inflammatory response. Taken together, these findings suggest that ERN mitigates IMQ-induced psoriasis-like inflammation by suppressing NF-κB signaling and its downstream inflammatory mediators.

### 3.10 Activation of KEAP1-NRF2 pathway in animal models

**Figure 9:**
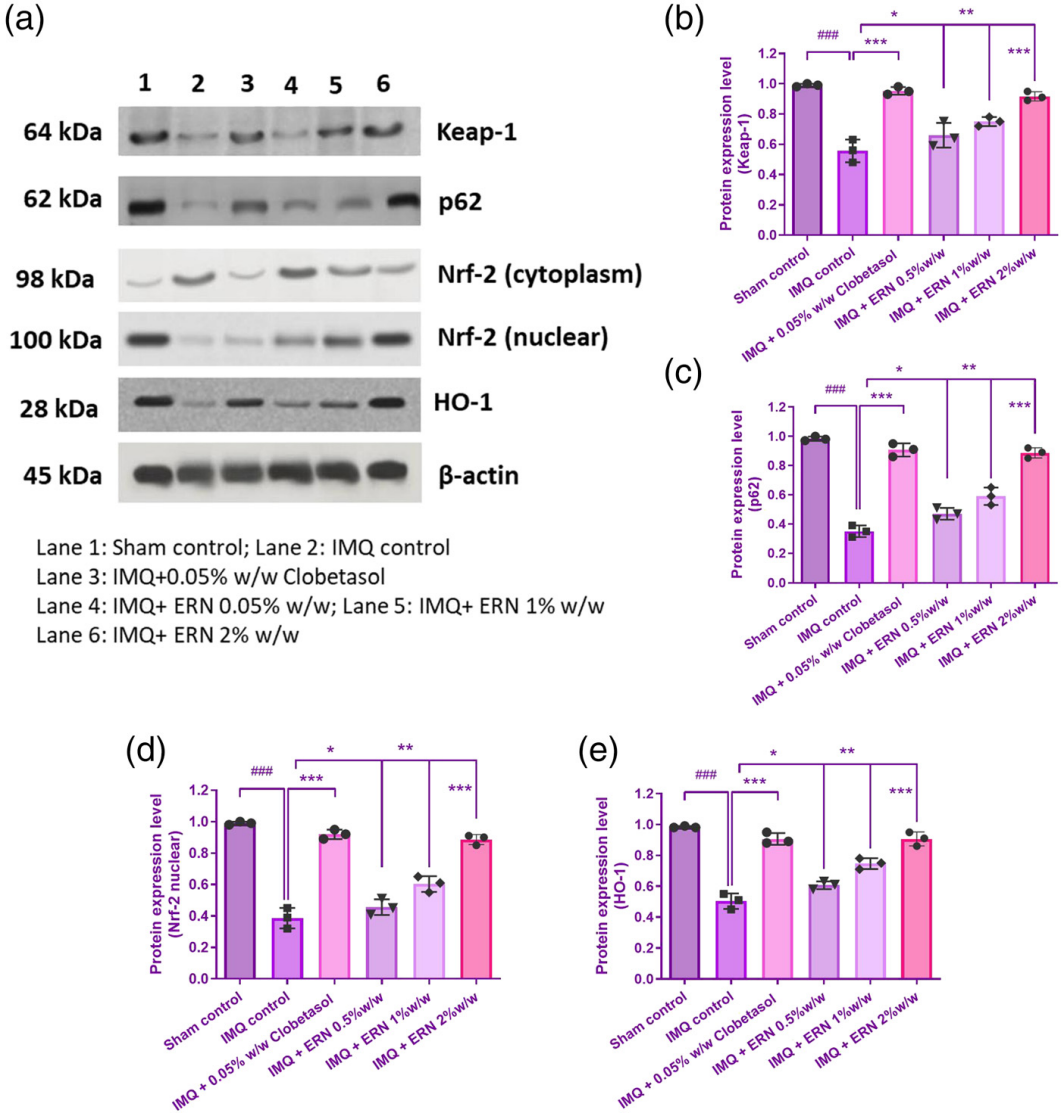
Impact of ERN therapy on the KEAP1-Nrf-2 signaling cascade in skin tissue that could be stimulated by IMQ. (a) The protein expression levels of Nrf2, p62, Keap1, and HO-1 in IMQ + ERN skin tissues are demonstrated by exemplary immunoblot studies. The internal control in this case was β-actin. (b)–(e) Shows various band intensities assessed by Image J software evaluation to protein expression levels graphically. Results are expressed as mean values ± SEM (*n* = 3). ^###^ *p <* 0.001, ^##^ *p <* 0.01, and ^#^ *p <* 0.05 compared with the sham control group; *p < 0.05, **p < 0.01, and ***p < 0.001 compared with the IMQ alone treated group; ns, non-significant.

The impact of ERN was then assessed by using immunoblotting techniques to influence the localization of HO-1 in the IMQ-induced skin and Nrf2 in the nuclear region. When comparing the IMQ-only treated group to the untreated control group, we discovered a substantial *p* < 0.001 drop in KEAP1 and nuclear deposition of NRF2 (Figure 9). On the contrary, western blot analysis demonstrated that ERN treatment expressively improved the levels of KEAP1, p62, HO-1, and NRF2 in the skin tissue (Figure 9a–e). Altogether, ERN progresses the antioxidant enzyme status in IMQ-induced psoriatic mice. These results propose that ERN modulates the KEAP1-NRF2 pathway *in vivo*, enhancing antioxidant defence mechanisms and reducing oxidative stress in psoriasis-like skin inflammation.

## 4 Discussion

Psoriasis is a long-term autoimmune skin disorder caused by an overreactive defense system and an increase in the number of epithelial keratinocytes. Additionally, there is now no effective treatment for this illness; nevertheless, there are several treatment possibilities provided to control its symptoms, such as topical therapies that mostly include steroid medications. Steroid usage over time is known to have detrimental effects, including heightened susceptibility to infections, liver damage, and diabetes. Consequently, the development of new and safer psoriasis therapy drugs is crucial. Natural bioactive compounds have a great probability for treating illnesses and may be better than synthetic ones [40,41].

The most notable component is ERN, a naturally occurring bibenzyl molecule that has been utilized in traditional Chinese medicine as an analgesic and antipyretic [31,42]. ERN has been successfully demonstrated to have a therapeutic impact on decreasing tumor development and angiogenesis both *in vivo* and *in vitro* [43,44,45]. It shares structural similarities with combretastatin A-4, an attractive candidate for reducing the aberrant angiogenesis of cancer growth [46,47]. The possibility of ERN as a psoriasis treatment has not yet been investigated.

Therefore, the current study sought to evaluate the potential of ERN as a therapeutic agent in psoriasis by assessing its impacts on critical inflammatory pathways, oxidative stress control, and keratinocyte proliferation. In an *in vivo* IMQ-induced psoriasis model, ERN effectively reduces psoriatic symptoms via modulating NF-κB signaling, inflammatory cytokine release, and antioxidant response processes. Previous investigations [25,48,49] have linked NF-κB and oxidative stress to the etiology of psoriasis, indicating that ERN might be a potential therapy option. Furthermore, current research supports the regulation of the KEAP1-NRF2 pathway shown in our work, stressing its function in reducing oxidative damage and inflammation in diverse skin diseases.

Strong evidence linking ERN’s effectiveness as a psoriasis treatment is provided by this investigation’s findings. As a persistent inflammatory skin condition, psoriasis is characterized by pro-inflammatory cytokines being produced and uncontrolled keratinocyte growth [50]. The capacity of ERN to inhibit the growth of psoriasis-like keratinocytes and the inflammatory mediator process, which is a defining feature of psoriasis pathophysiology, serves as one of the main conclusions of this study. This effect was shown in both *in vitro* and *in vivo* settings, representing that ERN has a wide-ranging impact on pathways linked to psoriasis. AKT/mTOR and JNK/c-Jun signaling driven by ROS inhibited the proliferation of HaCaT cells and resulted in their death [51].

ERN dramatically decreased the stimulation of these mechanisms, which subsequently in turn suppressed the formation of inflammatory molecules such as TNF-α, IL-6, IL-1β, IL-23, and IL-17, according to this investigation. It is important to note that IL-8 and IL-22 were only assessed in the cell-based experiments. IL-8, a major chemokine involved in neutrophil recruitment, is not functionally conserved in mice and thus was not included in the animal experiments. Similarly, IL-22, while relevant to skin inflammation, was not evaluated *in vivo* due to the prioritization of cytokines more commonly associated with the IMQ-induced murine model, such as IL-17 and IL-23. These choices reflect methodological differences and highlight how the *in vitro* and *in vivo* models offer complementary insights into the anti-inflammatory potential of the treatment. Current evidence suggests that these cytokines play a role in initiating and maintaining the inflammation associated with psoriasis [52,53]. ERN may aid in the restoration of immunological homeostasis in psoriasis by inhibiting these pathways. Furthermore, the ability of ERN to increase IL-10 levels, an anti-inflammatory, inflammatory substance, implies that ERN usually promotes an anti-inflammatory milieu. Especially, the excessive production of IL-10, which may aid in rebuilding and restoring the epidermal obstacles, are frequently compromised in psoriasis, in conjunction with inhibiting cytokines that cause inflammation.

Our findings highlight the need to combine *in vitro* and *in vivo* approaches for understanding ERN’s anti-psoriatic properties. The *in vitro* model focused on keratinocyte reaction to TNF-α stimulation, whereas the *in vivo* model assessed the overall immunological dynamics, such as the IL-23/IL-17 axis. This divergence across keratinocyte-centric *in vitro* inflammation and immune-driven *in vivo* responses emphasizes our study’s translational implications. It reveals how ERN has diverse immunomodulatory effects at many stages of the psoriatic inflammatory process. This comprehensive approach not only validates ERN’s effectiveness but also demonstrates its potential application across different phases and processes of psoriasis etiology.

It is critical to recognize the distinction between the IMQ-induced mouse model of psoriasis and human psoriasis. Although the IMQ model accurately reproduces several inflammatory aspects of psoriasis, such as epidermal hyperplasia, immune cell infiltration, and skin thickness, it does not entirely imitate the complicated immunopathogenesis seen in actual psoriasis. Human psoriasis is predominantly caused by the IL-23/Th17 axis, with Th17 cells generating pro-inflammatory cytokines including IL-17 and IL-22, which play an important role in inflammation and tissue destruction [54]. Conversely, the IMQ-induced psoriasis paradigm primarily stimulates the IL-1 and TLR7 pathways, which, while contributing to inflammation, vary from the main IL-23/Th17-mediated inflammation reported in patients [55,56]. Consequently, while the results we obtained give useful insights into the potential therapeutic benefits of ERN, more advanced models that better mirror the actual disease pathophysiology are required to properly comprehend the therapeutic impact of ERN in human psoriasis.

The observed reduction in IL-23 and IL-17 *in vivo* may be due to ERN-mediated NRF2 activation, as evidenced by enhanced nuclear Nrf2 and HO-1 expression in psoriatic lesions. Previous investigation has connected NRF2 activation to the suppression of Th17-related cytokines via redox-sensitive pathways [15,57]. However, the current approach did not show a clear causal association, our findings indicate that the NRF2 pathway may play a role in controlling Th17-driven inflammation in psoriasis. Further research using NRF2-specific inhibition or gene knockdown will assist in elucidating this process.

ERN effectively decreased the histological characteristics of psoriasis, which includes excessive keratin and epidermis dimension, along with its clinical signs, characterized as erythema and scaling, in the context of preliminary animal experiments. A rise in blood indicators, namely, a drop in WBC count, additionally showed the comprehensive anti-inflammatory effects of ERN, which may eventually result in therapeutic advantages in individuals.

The organ index, also known as the spleen-to-body mass ratio, was used to quantify total inflammation in the IMQ-induced psoriasis theory. Spleen enlargement is often associated with psoriasis and other inflammatory diseases because of increased immune cell trafficking and immunological activation. A normalized spleen-to-body weight connection in grams per gram (g/g) [38], was obtained by calculating the organ index in the current research by matching the weight of a particular animal’s spleen to its entire weight. The organ index (spleen-to-body mass proportion) findings demonstrate that ERN treatment significantly reduces spleen enlargement associated with IMQ-induced systemic inflammation. This reduction in the spleen index supports ERN’s role as an anti-inflammatory agent, potentially beneficial in alleviating both local and systemic inflammatory responses in psoriatic conditions.

Multiple investigations indicate that oxidative stress plays a foremost part in the pathophysiology of psoriasis. Reduced activity of anti-oxidant enzymes, augmented ROS levels, and an imbalance between oxidants and antioxidants are particular factors that impact the etiology and development of psoriasis. Ma et al. [28], Pleńkowska et al. [11], Bakić et al. [58], and Klisic et al. [59] suggest that oxidative stress biomarkers can be used to measure the severity of psoriasis, predict the course of other medical problems, and assess the effectiveness of treatment. Some of the exogenous oxidative stressors that can exacerbate psoriasis symptoms include medications, alcohol, smoking, stress, and bacterial infections [60–62]. Our study confirmed that ERN has antioxidant properties, as it significantly reduced the oxidative stress damage associated with psoriasis.

The KEAP1/NRF2 cascade has anti-inflammatory and antioxidant activity properties and is crucial for maintaining skin homeostasis [29]. Research indicates that NRF2 may enhance keratinocyte proliferation and aid in the formation of skin epidermal barriers [63,64]. Dimethyl fumarate, an NRF2 activator, is used therapeutically to treat moderately severe and mild psoriasis [65–67], and some of its constituents have been investigated and proposed to relieve psoriasis [68]. However, excessive NRF2 stimulation might be detrimental. Consequently, additional study is needed to understand how NRF2 impacts psoriasis and to identify safe and effective NRF2 stimulants for psoriasis treatment.

The primary process behind ERN’s anti-inflammatory and antioxidant actions is its stimulation of the KEAP1-NRF2 cascade. Under normal conditions, NRF2 binds to KEAP1 to maintain its inactive state in the cytoplasm. NRF2 separates from KEAP1 and moves to the nucleus in response to signs of oxidative stress or inflammation. There, it triggers the production of protective enzymes that lessen cell damage [28,69]. In our investigation, we found that ERN therapy increased the expression of NRF2 and its subsequent targets, including having an impact on reducing oxidative stress and deactivating ROS, including SOD, CAT, and GST. This implies that ERN may lessen the pathophysiology of psoriasis by regulating inflammation and lowering oxidative stress in the skin. The present investigation supports earlier findings [28]. However, our observations suggest that the therapeutic benefits of ERN may not follow a precise linear dosage response trend. For instance, the 1% ERN dosage exceeded the 2% dose in certain outcome measures, such as skin scaling and antioxidant enzyme activity. This shows that raising the dose over a particular threshold may not increase efficacy correspondingly, emphasizing the necessity of determining the ideal therapeutic range. Additional research is needed to understand the pharmacodynamic (PD) ceiling of ERN and find the most effective and safe dose range. Such nonlinear dose-response relationships have been reported in other natural compounds, including curcumin and resveratrol, where high doses do not always equate to greater efficacy and may, in some cases, reduce bioactivity [70,71].

Evidence indicates that NRF2 may support the development of skin-epidermal obstacles and enhance cell differentiation [20,66,72]. The NRF2 stimulator dimethyl fumarate has been employed therapeutically to treat moderately severe psoriasis [73], and some of its derivatives may be able to alleviate psoriasis. On the other hand, activating NRF2 too much could be harmful [19]. Thus, more investigation into how NRF2 functions in psoriasis is required, as is the hunt for safe and efficient NRF2-activating drugs to treat psoriasis.

Our results are in line with the existing research, which indicates that healthy skin has high levels of NRF2 expression [19,74,75]. Many skin conditions may benefit from NRF2 activation as a treatment. Remarkably, we discovered that in the lesional skin of a rat model of psoriatic IMQ, NRF2 expression was markedly decreased. ERN therapy significantly raised NRF2 expression and decreased the psoriatic pathogenic phenotype by halting oxidative stress-induced skin tissue damage and inflammation. Since NRF2 depletion increased the mice’s inflammatory reaction, aberrant keratinocyte development, and psoriasis-like signs and symptoms, the curative properties of ERN appeared to be NRF2-dependent. This result confirms our theory that ERN operates through NRF2-related pathways and that NRF2 protects against the onset of psoriasis.

According to Silva et al. [76], the popular inflammatory cytokine TNF-α is the cause of numerous inflammatory diseases. Macrophages are the primary generators of this toxic material during inflammation [77,78]. The main way that TNF-α causes inflammation is by paracrine and autocrine stimulation of macrophages, which increases the production of inflammatory cytokines like IL-1β, IL-6, COX-2, and iNOS. This can result in a cascade caused by an inflammation cascade [79–81]. Moreover, anti-TNF-α treatment alleviates dermatitis, and TNF-α contributes to the inflammatory process of skin inflammatory diseases [82,83].

Additionally, ERN increased the levels of antioxidant enzymes while dramatically lowering nitrites and TBARS. In the reaction to IMQ-induced psoriasis, these results led to the defensive activity of ERN by increasing antioxidant enzymes, preventing the production of lipid peroxidation byproducts in macrophages via COX-2 expression, and lowering nitrite concentrations within iNOS expression. ERN-treated animals with IMQ-induced psoriasis showed markedly decreased amounts of COX-2 and iNOS protein expression. By activating the Nrf2 system in psoriatic mice, we explain how the number of plant-based constituents and associated structural analogues provide increased protection against oxidative stress [38,84]. The increase in phase II-detoxifying and antioxidant enzymes from oxidative damage is aided by Nrf2 modulation [85]. According to a prior study, Nrf2 expression interruptions used redox control to repress the pro-inflammatory and inflammation-related genes of mediators [86–88]. The impact of ERN on the pathway used by NF-B to signal in skin tissues was confirmed by our study. By improving the expression of KEAP1, p62, and HO-1, we additionally investigated how ERN effectively increased the nuclear translocation of NRF2. Thus, ERN protects against psoriasis caused by IMQ through the antioxidant enzyme’s defense mechanism, which is aided by KEAP1-Nrf2.

Dimethyl fumarate (DMF), a well-known NRF2 activator approved for clinical use in treating psoriasis and multiple sclerosis, has demonstrated significant efficacy by modulating oxidative stress and immune responses through NRF2 pathway activation [66,89]. However, DMF is often associated with side effects such as gastrointestinal discomfort, flushing, and lymphopenia, which may impact long-term patient adherence [90].

In contrast, ERN, as evidenced by our findings, exhibits strong antioxidant and anti-inflammatory properties by activating the KEAP1-NRF2 pathway, with potentially fewer adverse effects. Notably, preclinical studies suggest that ERN can suppress key pro-inflammatory mediators and restore redox homeostasis with high potency [91]. While clinical trials are still lacking, the favorable safety and efficacy profiles observed in our study suggest that ERN could be a promising alternative to traditional NRF2 activators like DMF. Comparative studies assessing efficacy, bioavailability, and safety are essential to validate ERN’s potential advantages in a clinical setting.

Although ERN displayed strong anti-inflammatory and antioxidant benefits in both *in vitro* and *in vivo* psoriasis models, it is crucial to note that the exact pharmacokinetic (PK) and PD profiles of ERN are still poorly understood. Existing preclinical studies show that ERN has low water solubility and is rapidly degraded, potentially limiting its bioavailability and systemic exposure. These qualities may limit its clinical applicability until addressed by formulation tactics such as nanoparticle delivery systems or prodrug approaches. Furthermore, no human clinical studies or thorough PK/PD investigations have been published thus far. Future research should focus on complete PK analysis and delivery system optimization to fully investigate ERN’s therapeutic potential and support its clinical adoption.

In addition, whereas our work demonstrates the beneficial effects of ERN on inflammation and oxidative stress, the long-term ramifications of ERN, notably chronic NRF2 activation, must be carefully considered [92,93]. NRF2 is essential in cellular responses to oxidative stress, but prolonged activation of NRF2 may have unexpected repercussions, such as changes in immune cell function or disruption of normal cellular processes in other organs. Future studies should extensively study these possible consequences to deeply comprehend ERN’s safety profile. Long-term studies are required to determine the chronic consequences of NRF2 activation *in vivo*, as well as the influence on immune cells and diverse organs, in order to ensure ERN’s safety for long-term usage in clinical settings.

## 5 Conclusion

The current investigation shows that ERN has substantial potential as a treatment agent for psoriasis-like lesions. ERN reduces psoriasis symptoms by altering key molecular mechanisms, such as the KEAP1-NRF2 signaling cascade, which is important for lowering inflammation and oxidative stress. ERN has been shown to diminish TNF-α-induced inflammation and keratinocyte hyperproliferation in both *in vitro* and *in vivo* models, indicating it could serve as a reliable and efficient treatment for psoriasis. However, it is crucial to emphasize that, though the IMQ-induced psoriasis model gives useful insights into the disease process, the pathophysiology of clinical psoriasis may vary. To completely grasp the therapeutic potential of ERN in human psoriasis, more research with clinical samples is required. These investigations will serve to confirm ERN’s clinical relevance and influence future psoriasis therapy alternatives.

## Supplementary Material

Supplementary Figure
