# Supplementary material for: Modulation of the KEAP1-NRF2 pathway by Erianin: A novel approach to reduce psoriasiform inflammation and inflammatory signaling
Source: Open Life Sci. 2025 Jul 11;20(1):20251139. doi: 10.1515/biol-2025-1139 (PMC12260353; doi:10.1515/biol-2025-1139)
Supplement: Supplementary Figure [file biol-2025-1139-sm.pdf]

Supplementary material

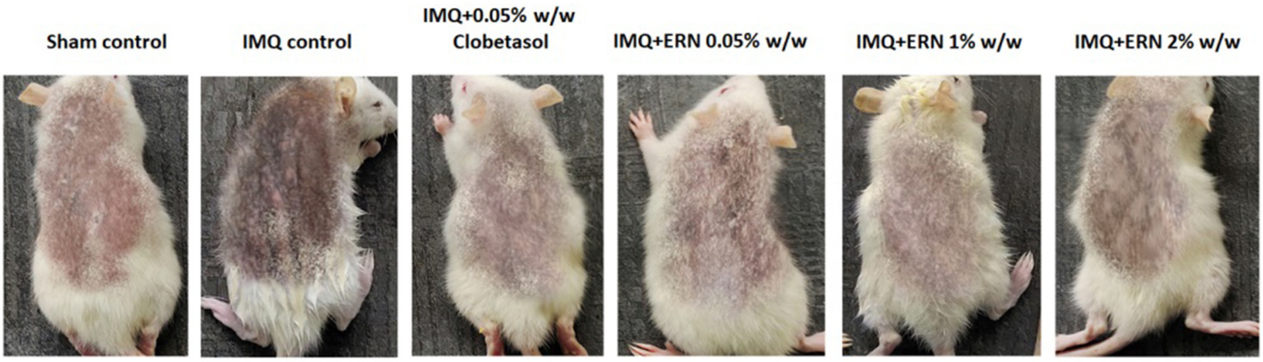

Figure S1: Representative images of dorsal skin from mice in different treatment groups during the experimental period day 6 showing visible morphological changes characteristic of psoriasis-like lesions, including erythema, scaling, and thickening. IMQ-treated mice exhibit pronounced erythema and thickened; scaly skin compared to the sham control. Treatment with ERN and clobetasol resulted in notable improvement in skin morphology.
